# Quality of Life in Mothers With Perinatal Depression: A Systematic Review and Meta-Analysis

**DOI:** 10.3389/fpsyt.2022.734836

**Published:** 2022-02-15

**Authors:** Jiaying Li, Juan Yin, Ahmed Waqas, Zeyu Huang, Hongji Zhang, Manqing Chen, Yufei Guo, Atif Rahman, Lei Yang, Xiaomei Li

**Affiliations:** ^1^School of Nursing, Xi'an Jiaotong University, Xi'an, China; ^2^School of Nursing, Dalian University, Dalian, China; ^3^Institute of Population Health, University of Liverpool, Liverpool, United Kingdom

**Keywords:** health-related quality of life, quality of life, perinatal depression, postpartum depression, antepartum depression

## Abstract

**Background:**

The prevalence of perinatal depression is high and its adverse effects on mothers and infants are extensive. Several studies have explored the relationship between perinatal depression and health-related quality of life (HRQoL), but little is known about the nature and magnitude of this effect. The objectives of this study were to evaluate the HRQoL of mothers with perinatal depression and compare the HRQoL of depressed mothers with that of non-depressed mothers.

**Methods:**

A systematic review was performed according to the PRISMA guidelines. PubMed, EMBASE, Scopus, PsycINFO, Web of Science, Cochrane Central Register, the China National Knowledge Infrastructure, the VIP Database, and the Wan Fang Database were searched. The retrieval time was from the establishment of the database to July 2020. A series of meta-analyses were run for each outcome pertaining to HRQoL sub-measures. Subgroup analyses were conducted based on country income category and time period.

**Results:**

Of 7,945 studies identified, 12 articles were included in the meta-analysis, providing HRQoL data for 4,392 mothers. Compared with non-depressed mothers, mothers with perinatal depression reported significantly poor scores across all the quality-of-life domains. Mixed-effects analysis showed that there was no difference in the HRQoL scores of mothers with antepartum and postpartum depression. Mothers with perinatal depression in higher-income countries reported higher disability on role-physical (*p* = 0.02) and social functioning domains (*p* = 0.001) than those from lower-income countries.

**Limitations:**

Due to insufficient data, no regression analysis was performed. The inability to accurately determine the difference in HRQoL between antepartum and postpartum depression was because of the restriction of the included studies. Moreover, most of the included studies were conducted in middle-income countries and may have an impact on the applicability of the results. Subgroup analyses are observational and not based on random comparisons. The results of subgroup analyses should be interpreted with caution.

**Conclusion:**

HRQoL is compromised in mothers with perinatal depression. Continuous efforts are required to improve the HRQoL of perinatal depressed mothers.

**Systematic Review Registration:** CRD42020199488.

## Introduction

Perinatal depression occurs during pregnancy or within 1 year after delivery (1) with its prevalence estimated at 10–15% in high-income countries (HIC) and 15–25% in low and middle-income countries (LMICs) (2–4). During the perinatal period, many biochemical, physiological and anatomical changes occur in the mother's body (5). These changes lead to depression and other negative emotions, which are harmful to the physical and mental health of perinatal mothers (6), thus affecting their health-related quality of life (HRQoL). Therefore, HRQoL has become an important indicator of perinatal health evaluation (7).

Perinatal depression is associated with an array of adverse effects both in mothers and children. Among pregnant and postpartum mothers, perinatal depression is related to reduced social support and self-care, increased substance use, work-related dysfunction, malnutrition, and other obstetric complications (8–10). Among children born to perinatal depressed mothers, there is a higher likelihood of developing physical development problems, including premature birth and low birth weight, low immunization rates, high incidence of infectious diseases (e.g., diarrhea), as well as cognitive and socioemotional development problems, such as cognitive delay, negative child temperament, oppositional behavior, and attention deficit symptoms (11–14).

Previous literature has shown an indirect relationship between quality of life and perinatal depression. However, there is a paucity of literature on different constructs and domains of quality of life that are the most adversely affected. Therefore, the aims of this systematic review were to evaluate the HRQoL of mothers with perinatal depression and compare the HRQoL of depressed mothers with that of non-depressed mothers. This review is intended to fill the gap of previous studies, clarify and quantify the impact of perinatal depression on quality of life.

## Methods

This systematic review used PRISMA guidelines for conducting and reporting systematic reviews (15), and the protocol for this review was prospectively registered on PROSPERO (CRD42020199488, 17 August 2020). We systematically searched nine international and Chinese electronic databases to retrieve relevant articles: PubMed, EMBASE, Scopus, PsycINFO, Web of Science, Cochrane Central Register, the China National Knowledge Infrastructure, the VIP Database for Chinese Technical Periodicals, and the Wan Fang Database for Chinese Periodicals, key search terms included “Perinatal,” “Depression” and “Health-related quality of life,” from the inception of databases through July 2020. The complete search strategy was shown in Appendix 1.

The most commonly used HRQoL scales during the perinatal period include the Medical Outcomes Study Short Form 36-Item / 12-Item Health Survey (SF-36 and SF-12) and the World Health Organization Quality of Life assessment-bref (WHOQOL-BREF). SF-36 has 36 items, measuring eight domains of health concepts, namely physical functioning, role-physical, bodily pain, general health, vitality, role-emotional, social functioning and mental health. The scores of the above eight subscales are further classified into two categories: physical component score (PCS) and mental component score (MCS). The higher the score (from 0 to 100), the better the HRQoL (16). SF-12 is a validated abbreviated version of the 36-item Survey. WHOQOL-BREF produces scores in four domains pertaining to HRQoL: physical health, psychological, social relationships and environment. The potential range of scores for all domains is 4–20, higher scores indicate better quality of life (17).

For review, we included cross-sectional and longitudinal (retrospective and prospective) studies which reported on different aspects of quality of life among mothers with perinatal depression. We defined the perinatal period as starting from pregnancy to 1 year after delivery and perinatal depression was defined according to diagnostic interview based on clinical criteria (e.g., DSM-5 or ICD-11), or as a score above a cut-off on a self-rating depression scale (e.g., PHQ-9, SDS, HAMD, etc.). For measurement of quality-of-life outcomes, we considered all those studies that used a validated self-report scale such as but not limited to SF-36 and WHOQOL-BREF. The exclusion criteria included studies focusing on participants with comorbid physical diseases or medical conditions (e.g., gestational diabetes mellitus, hypertension, etc.). We also excluded those studies that report only a part or subscale score of a validated measure of HRQoL. We also excluded articles published in languages other than English or Chinese; Books, book sections, patents, qualitative studies, review articles, conference abstracts and protocol.

After removing duplicate articles using EndNote, two reviewers (MC and YG) independently screened the titles and abstracts for eligibility, followed by the full-text screening. Finally, the list of articles for inclusion was cross-checked between authors, and any discrepancies were resolved by a senior reviewer (JL).

## Data Extraction and Meta-Analysis

Two authors extracted the data from studies, using a piloted excel sheet, working independently from each other. General information of publications and research design were extracted, including study design, country of study, geographical scope (urban, rural or semi-urban), setting of study (hospital, community, etc.), comparison groups (if any), year of publication, and the outcome measures including the type of diagnostic or screening instruments utilized to ascertain perinatal depression and measures for HRQoL. Quantitative data for meta-analysis to evaluate and compare HRQoL such as sample size, scores of depression in each group, each and total HRQoL scores of each group at different periods. The data were cross-checked between the two authors (JL and HZ) and any inconsistencies were resolved through discussion with a third author (JY).

The methodological quality of each article was evaluated independently by two reviewers (JL and ZH) and any discrepancies were resolved by a third reviewer (JY). Based on the types of included studies, different quality assessment tools were selected. For cohort and case-control studies, the risk of bias was assessed using the Newcastle-Ottawa quality assessment scale (18). Studies with scores of five points or more were considered to be of moderate to good study quality (19). For cross-sectional studies, the quality was assessed using a checklist from the Joanna Briggs Institute (20).

A series of meta-analyses were run for each outcome pertaining to the quality-of-life sub-measures. Mean (SD) related to the quality-of-life sub-measures were extracted for healthy mothers and mothers with perinatal depression. These quantitative data were used to calculate correlation coefficients and their 95% confidence intervals and then pooled using the random effects with DerSimonian & Laird method. Random effects were employed throughout the analyses due to expected clinical and statistical heterogeneity. This inconsistency across studies was quantified using the I^2^ statistic considered significant at 40%. Sensitivity analyses using the knockout approach were performed to check the contribution of individual studies to the pooled effect size. Publication bias was assessed by visualizing Begg's funnel plot and statistically using Egger's regression statistic considered significant at *p* > 0.1. If significant publication was evident, Duval & Tweedie's trim and fill method was used to impute studies to yield effect sizes adjusted for the publication bias.

Subgroup differences based on country income category and time period (antepartum vs. postpartum) were run using mixed-effects analyses. Meta-regression analyses were not run due to a lack of statistical power, owing to the fewer number of studies reporting each outcome.

## Result

**Figure 1:**
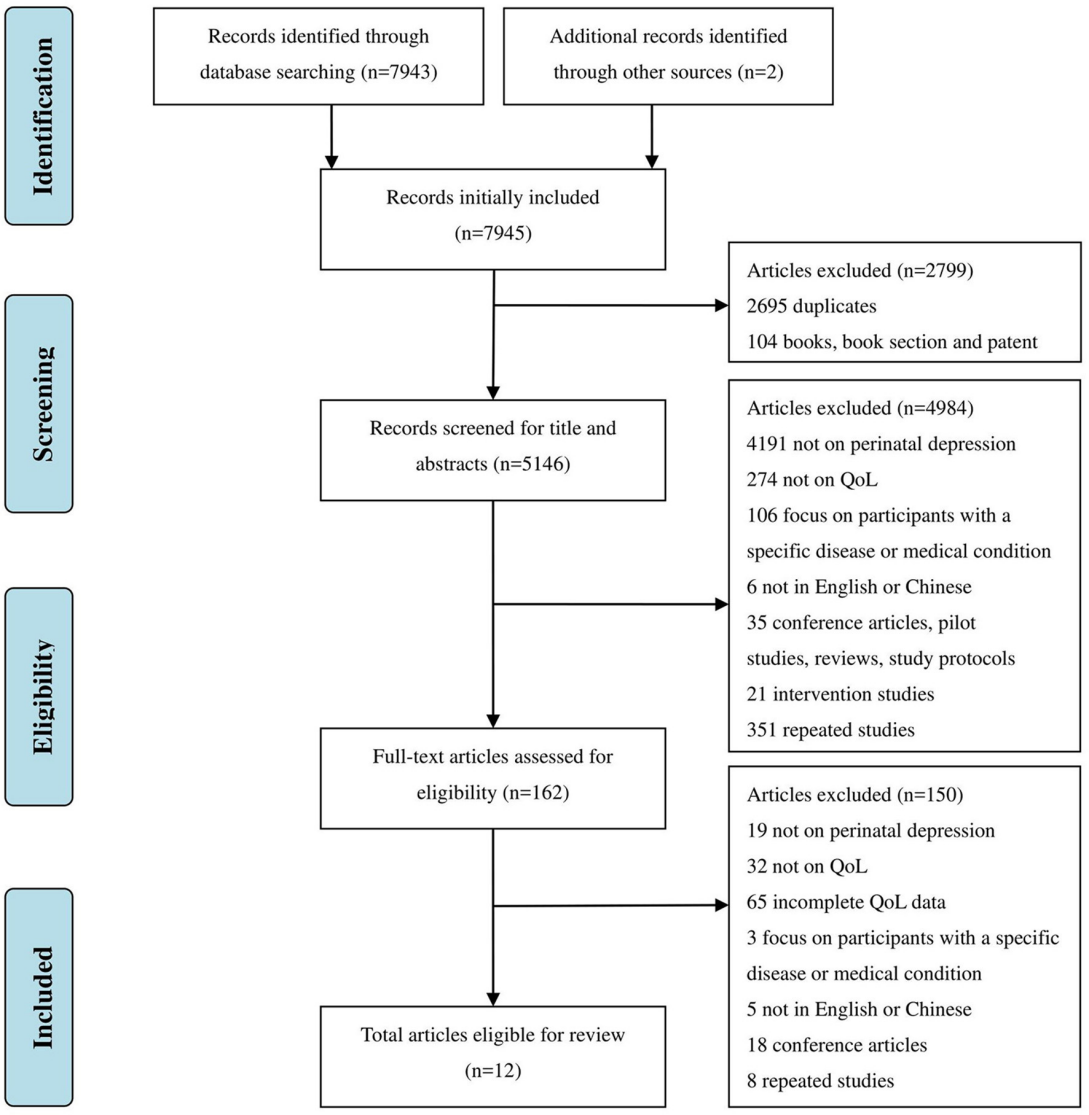
Study selection process. QoL, quality of life.

A total of 7,943 articles were identified after systematically searching 9 databases. Another 2 articles met the inclusion criteria through the screening of bibliography from included articles. After removal of books, book sections, patents (104) and duplicate articles (2,695), a further 4,984 articles were excluded through screening of title and abstract. This led to 162 articles entering the full-text search stage. At this stage, a further 150 articles were excluded. Finally, 12 articles were included in this review (Figure 1).

For quality assessment, after preliminary assessment by two independent reviewers, the agreement between items was 97.3%, all differences were resolved in the consensus meeting with the senior author (Appendix 2). The scores of the three case-control and cohort studies were all 8, indicating a low risk of bias (21–23). When assessing cross-sectional studies, three items were ignored by more than two studies: the clear definition of inclusion criteria and the identification/treatment of confounding factors, which may lead to research bias in these aspects.

Quality of life was assessed in a total of 4,392 perinatal mothers, including 1,151 pregnant or postpartum mothers with depression and 3,241 healthy controls. Of the 12 studies included, 9 were cross-sectional studies, while the rests were case-control and cohort studies. The included studies were carried out in six countries, including China, Iran, America, France, Nigeria and Canada. Among these, 5 studies were conducted antenatally and 7 in the postpartum period. Among the 12 studies examining HRQoL, 11 studies used SF-36 and only 1 study utilized WHOQOL-BREF (Table 1).

**Table 1:** Studies characteristics (*N* = 12).

**References**	**Design**	**Subject**	**Sample size**	**Age (Mean ± SD) years**	**Time**	**Country**	**Scale**	**Sampling method**
Li et al. (44)	CS	Depressed (EPDS≥9.5); Non-depressed (EPDS <9.5)	181; 273	28.5	Antepartum	China	SF-36	Non-randomized
Chen (45)	CS	Depressed (EPDS>9.5); Non-depressed (EPD ≤ 9.5)	119; 181	18–40	Antepartum	China	SF-36	Non-randomized
Da Costa et al. (24)	CS	Depressed (EPDS≥10); Canadian norm	78; 198	19–43 (33.17 ± 4.56); 25–34	Postpartum	Canada	SF-36	Non-randomized
de Tychey et al. (25)	CS	Severe depressed (EPDS≥12); Mild depressed (8 ≤ EPDS <12); Non-depressed (EPDS <8)	17; 40; 124	19–40 (29 ± 5)	Postpartum	France	SF-36	Non-randomized
Sadat et al. (21)	Cohort	Depressed (EPDS≥13); Non-depressed (EPDS <13)	75; 246	NR	Postpartum	Iran	SF-36	Randomized
Nicholson et al. (46)	CS	Depressed (CES-D≥16); Non-depressed (CES-D <16)	27; 148	27.3 ± 6.4; 28.8 ± 6.5	Antepartum	America	SF-36	Non-randomized
Abbaszadeh et al. (23)	Case-control	Depressed (BDI>9); Non-depressed (BDI ≤ 16)	112; 353	25.40 ± 4.63; 25.32 ± 4.41	Antepartum	Iran	SF-36	Non-randomized
Qiu (22)	Case-control	Depressed (EPDS>12); Non-depressed (EPDS ≤ 12)	70; 70	20–41 (25.1 ± 8.3); 20–40 (25.3 ± 8.5)	Postpartum	China	SF-36	Non-randomized
Hu and Lu (47)	CS	Depressed (EPDS≥13); Non-depressed (EPDS <13)	126; 851	17–42(28.32 ± 4.37)	Postpartum	China	SF-36	Non-randomized
Zhang et al. (48)	CS	Depressed (EPDS>12); Non-depressed (EPD ≤ 12)	169; 250	20–44 (29.9 ± 3.9)	Postpartum	China	SF-36	Non-randomized
Tsai (49)	CS	Depressed (EPDS≥13); Non-depressed (EPDS <13)	21; 132	32.6 ± 3.18	Antepartum	China Taiwan	SF-36	Non-randomized
Tungchama et al. (26)	CS	Depressed (EPDS≥12 and diagnosed by DSM-IV); Non-depressed (EPDS <12)	116; 415	18–45 (26.84 ± 5.6)	Postpartum	Nigeria	WHOQOL-BREF	Non-randomized

*CS, Cross-sectional; EPDS, The Edinburgh Postnatal Depression Scale; CES-D, The Center for Epidemiologic Studies Depression Scale; BDI, Beck Depression Inventory; DSM-IV, Diagnostic and Statistical Manual of Mental Disorders, fourth edition; NR, Not reported; SF-36, The Medical Outcomes Study Short Form 36-Item Health Survey; WHOQOL-BREF, World Health Organization Quality of Life assessment-bref*.

The outcomes pertaining to bodily pain, general health, mental health, physical functioning, role emotional and role physical and vitality were reported as an outcome in ten studies with a cumulative sample size of 3,686 mothers. The outcomes pertaining to social functioning were reported in 11 studies (*n* = 4217) and physical component score (PCS) and mental component score (MCS) in six studies each (*n* = 2063).

**Figure 2:**
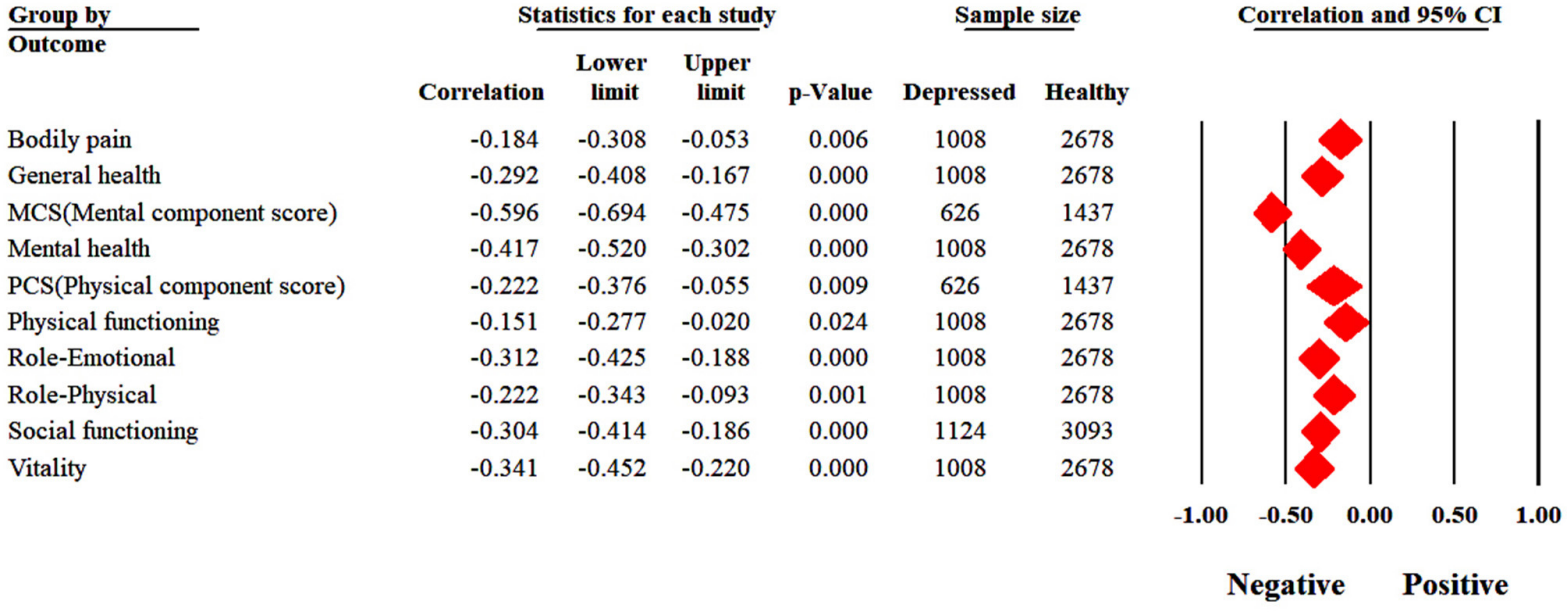
Forest plot.

All outcomes revealed substantial heterogeneity except bodily pain, general health and physical functioning. Mothers with perinatal depression reported significantly poor scores across all the quality-of-life outcomes (Figure 2). Mothers with depression reported greater problems with MCS (r = −0.60, *p* < 0.001); mental health (r = −0.42, *p* < 0.001); social functioning (r = −0.30, *p* < 0.001) and vitality (r = −0.34, *P* < 0.001) and role-emotional (r = −0.31, *p* < 0.001). These correlations suggested moderate strength of effect sizes. While outcomes related to physical health yielded weaker associations including bodily pain (r = −0.18, *p* = 0.006); general health (r = −0.29, *p* < 0.001); PCS (r = −0.22, *p* = 0.009); physical functioning (r = −0.15, *p* = 0.024), role-physical (r = −0.22, *p* = 0.001).

Publication bias was noted only in reporting of general health (*p* = 0.07), physical functioning (*p* = 0.01), role-physical (*p* = 0.07) and social functioning (*p* = 0.02). Funnel plots for all domains of quality of life can be seen in Appendix 3. Duval and Tweedie's trim and fill method yielded an adjusted correlation of −0.12 for physical functioning and −0.31 for social functioning.

According to different time periods, mixed-effects analyses showed no statistically significant subgroup differences in effect sizes on the different quality of life domains. Several statistical subgroup differences were noted based on income groups of countries. Mothers with perinatal depression in higher-income countries reported higher disability on role-physical and social functioning domains than their counterparts (Tables 2, 3).

**Table 2:** Subgroup analyses using mixed-effects according to time period (*N* = 11).

**Domain**	**Subcategory**	**Number of studies**	**Effect size (95% CI)**	**I^**2**^**	**Q value (*p*)**
Bodily pain	Antepartum	4	−0.21 (−0.26 −0.16)	90.5%	1.8 (0.18)
	Postpartum	6	−0.16 (−0.20 −0.12)	73.3%	
General health	Antepartum	4	−0.28 (−0.32 −0.23)	0%	0.18 (0.67)
	Postpartum	6	−0.30 (−0.37 −0.22)	72.29%	
MCS	Antepartum	2	−0.84 (−0.98–0.003)	99.73%	1.65 (0.20)
	Postpartum	4	−0.39 (−0.47 −0.31)	66.51%	
Mental health	Antepartum	4	−0.48 (−0.54 −0.42)	51.69%	3.93 (0.05)
	Postpartum	6	−0.37 (−0.46 −0.27)	85.59%	
PCS	Antepartum	2	−0.18 (−0.25 −0.12)	0%	0.43 (0.52)
	Postpartum	4	−0.24 (−0.39 −0.08)	89.53%	
Physical health	Antepartum	4	−0.14 (−0.20 −0.09)	0%	0.03 (0.86)
	Postpartum	6	−0.14 (−0.18 −0.09)	12.87%	
Role-emotional	Antepartum	4	−0.31 (−0.44 −0.17)	87.87%	0.001 (0.98)
	Postpartum	5	−0.31 (−0.40 −0.21)	83.29%	
Role-physical	Antepartum	4	−0.23 (−0.35 −0.12)	80.58%	0.13 (0.72)
	Postpartum	6	−0.21 (−0.29 −0.12)	78.27%	
Social functioning	Antepartum	4	−0.28 (−0.33 −0.24)	0%	0.26 (0.61)
	Postpartum	7	−0.31 (−0.38 −0.23)	77.59%	
Vitality	Antepartum	4	−0.33 (−0.38 −0.29)	2.86%	0.03 (0.87)
	Postpartum	6	−0.34 (−0.44 −0.24)	86.82%	

*MCS, Mental component score; PCS, Physical component score*.

**Table 3:** Subgroup analyses using mixed-effects according to income group of countries (*N* = 11).

**Domain**	**Subcategory**	**Number of studies**	**Effect size (95% CI)**	**I^**2**^**	**Q value (*p*)**
Bodily pain	HIC	2	−0.18 (−0.30 −0.06)	43.90%	0 (0.10)
	MIC	8	−0.18 (−0.21 −0.15)	0%	
General health	HIC	2	−0.29 (−0.43 −0.15)	64.24%	0.01 (0.91)
	MIC	8	−0.28 (−0.33 −0.23)		
MCS	HIC	2	−0.46 (−0.53 −0.39)	0	0.43 (0.51)
	MIC	4	−0.65 (−0.92–0.03)	99.7%	
Mental health	HIC	2	−0.47 (−0.54 −0.40)	0%	1.71 (0.19)
	MIC	8	−0.41 (−0.48 −0.32)	86.88%	
PCS	HIC	2	−0.15 (−0.23 −0.05)	0%	1.84 (0.17)
	MIC	4	−0.26 (−0.25 −0.10)	89.05%	
Physical health	HIC	2	−0.19 (−028 −0.10)	0%	1.68 (0.20)
	MIC	8	−0.13 (−016 −0.10)	0%	
Role-emotional	HIC	2	−0.33 (−0.44 −0.21)	51.79%	0.14 (0.71)
	MIC	8	−0.31 (−0.39 −0.22)	86.48%	
Role-physical	HIC	2	−0.32 (−0.40 −0.24)	0%	5.07 (0.02)^*^
	MIC	8	−0.19 (−0.26 −0.12)	76.68%	
Social functioning	HIC	2	−0.42(−0.49 −0.34)	0%	11.35 (0.001)^*^
	MIC	9	−0.27 (−0.31 −0.23)	41.64%	
Vitality	HIC	2	−0.45 (−0.58 −0.29)	76.07%	2.76 (0.10)
	MIC	8	−0.31 (−0.37 −0.25)	67.28%	

* *Significantly difference, MCS, Mental component score; PCS, Physical component score; HIC, High-income countries; MIC, Middle-income countries*.

For case-control and cohort studies, Abbaszadeh et al. (23) and Sadat et al. (21) gained full scores (9). Qiu et al. (22) yielded a score of 8, bias was observed to be due to the representativeness of the cases. For cross-sectional studies, biases mainly stemmed from the unspecific description of inclusion criteria, study subjects and the setting, and the improper identification and handling of confounding factors.

In total, 3 studies described factors associated with depressed mothers' HRQoL (24–26). Two studies assessed whether the gender of the child had an impact on HRQoL scores. Tychey et al. found that the baby's gender (having a boy) significantly reduced the quality of life. While Tungchama et al. found that that the baby's gender and spouse's expected gender of the infant were not associated with HRQoL scores.

Two studies investigated the impact of complications and mode of delivery on HRQoL. The results of one study showed that complications during pregnancy and delivery by cesarean were related to worse mental health status (24). Another study found that these two variables can reduce scores for social relationships and physical health domains, respectively (26). Costa et al. reported an association between greater cardiovascular fitness and better physical health status. The study also found lower physical health scores in multiparous mothers. As for the age of the study participants, one study found worse mental health scores in mothers younger than 35 (26). One study reported that poor sleep quality, poor social support and life stress can damage mental health (24).

## Discussion

This systematic review indicates that compared with non-depressed mothers, pregnant and postpartum mothers with perinatal depression have lower HRQoL scores, highlighting the profound impact of perinatal depression on their quality of life. This negative impact of depressive symptoms stays consistent across the antepartum or postpartum period. Mothers from the Western nations show more impairment in social functioning and role-physical domains of HRQoL than their counterparts.

There are several reasons for mothers with perinatal depression to have a poor HRQoL. First of all, Studies have found that there are abnormalities in the Hypothalamic-pituitary-adrenal (HPA) axis in patients with depression (27, 28). The activated HPA axis can not only regulate the body's peripheral functions such as metabolism and immunity but also play a linking role between stress and brain function (29, 30). Similarly, perinatal depression is also related to the excessive activation of the HPA axis, which may lead to alterations in the mother's physical and psychological functions, thereby affecting the quality of life (31, 32). Another possible explanation for the lower quality of life scores in mothers with perinatal depression may be the chronic nature of the disease. In most poor-resource settings, due to human resource constraints and ill-equipped medical systems, perinatal depression is largely undiagnosed and untreated, and as many as 90% of mothers with perinatal depression are not treated (33). Untreated depression usually becomes chronic and has a negative impact on the quality of life (34, 35).

Although mothers with perinatal depression reported significantly poor scores across all the quality-of-life outcomes, mental health appeared to be more affected by depression. For the MCS domain, a much lower score is indicative of frequent mental distress, as well as social and role disabilities due to emotional problems (36). It reminds family members and medical staff to provide mothers with more spiritual and life support, to help mothers recognize and overcome their unhealthy emotions, so as to improve the quality of life (37).

We found that there are differences in role-physical and social functioning between HIC and middle-income countries (MIC), with the lower scores reported in HIC. This result is inconsistent with the research result done by Lagadec et al., whose research believes that the absence of economic problems is strongly related to a better quality of life (38). However, in addition to economic reasons, the family system may also have an impact. Mothers in Eastern cultures tend to have tighter family connections, and most mothers and young children live in multi-generational family systems. Other family members (such as experienced grandmothers) are often involved in maternal care as well as infant feeding (39). Senior and more experienced women often play a vigorous role in caring for mothers, enabling them to be in better physical condition to perform work and other daily activities, that is, to function in better physical roles. Moreover, mothers generally report that when they are doing housework, taking care of their children, being physically and mentally unwell, grandmothers will participate and support them, alleviate their physical and emotional problems, which enable them to perform better social functions (40). Apart from the explanations above, these differences may also be explained by regional differences in living conditions, public health systems and perceptions of depression (41).

## Strengths and Limitations and Future Directions

To the best of our knowledge, this is the first systematic review to investigate HRQoL in mothers with perinatal depression. Through the inclusion of various types of research, sufficient data has been obtained for meta-analysis. However, our research has some limitations. Due to the small number and high heterogeneity of included studies, no regression analyses were carried out. And there was no longitudinal study continuously investigating the postpartum condition of mothers with antepartum depression. Therefore, we could not draw an accurate conclusion about the difference in the quality of life of depressed mothers before and after childbirth. In addition, most of the included studies were conducted in MIC, and economic level affects the quality of life (38), so the applicability of the results needs to be considered. Finally, the insights from the subgroup analyses should be interpreted with caution, due to the observational nature of this evidence.

Owing to the significant adverse effects of perinatal depression on mother's quality of life, it is necessary to improve the screening and treatment capabilities for perinatal depression. We, therefore, recommend the use of depression screening and HRQoL instruments in both research and clinical settings to detect perinatal depression and assess the HRQoL of mothers with perinatal depression. Besides, antepartum depression is a key risk factor for postpartum depression, leading to the persistence of depressive symptoms during the perinatal period (42, 43). So, studies are needed to continuously and actively assess depression and HRQoL before and after childbirth. Ultimately, findings pertaining to cultural differences in exhibiting different impairments in quality of life can also help in the design of more targeted and personalized interventions.

## Conclusion

This systematic review and meta-analysis reveal poorer HRQoL of mothers with perinatal depression as compared with non-depressed mothers, suggesting that perinatal depression has a significant adverse effect on HRQoL.

## Data Availability Statement

The original contributions presented in the study are included in the article/Supplementary Material, further inquiries can be directed to the corresponding author.

## Author Contributions

JL, JY, LY, AR, and XL: conception and design. JL, ZH, HZ, MC, and YG: collection and assembly of data. AW, JL, and JY: data analysis and interpretation. JL: substantial contributions to the first draft of the article. JL, AW, XL, and JY: work on the revised version of the manuscript. All authors approved the final manuscript.

## Funding

This study was funded by Interdisciplinary and Industry Fund, University of Liverpool, Cost Center JXR13462. The funder of the study had no role in the study design, data collection, data analysis, data interpretation, or writing of the report. The corresponding author had full access to all the data in the study and had final responsibility for the decision to submit for publication.

## Conflict of Interest

The authors declare that the research was conducted in the absence of any commercial or financial relationships that could be construed as a potential conflict of interest.

## Publisher's Note

All claims expressed in this article are solely those of the authors and do not necessarily represent those of their affiliated organizations, or those of the publisher, the editors and the reviewers. Any product that may be evaluated in this article, or claim that may be made by its manufacturer, is not guaranteed or endorsed by the publisher.

## Abbreviations

HRQoL: health-related quality of life
HIC: high-income countries
LMICs: low and middle-income countries
MIC: middle-income countries
SF-36/12: The Medical Outcomes Study Short Form 36/12-Item Health Survey
WHOQOL-BREF: World Health Organization Quality of Life assessment-bref
PCS: physical component score
MCS: mental component score
DSM-5: Diagnostic and Statistical Manual of Mental Disorders, fifth edition
ICD-11: International Classification of Diseases-11
SDS: Self-Rating Depression Scale
HAMD: Hamilton Depression Scale
PHQ-9: The Patient Health Questionnaire.
