# Supplementary material for: Quality of Life in Mothers With Perinatal Depression: A Systematic Review and Meta-Analysis
Source: Front Psychiatry. 2022 Feb 15;13:734836. doi: 10.3389/fpsyt.2022.734836 (PMC8886107; doi:10.3389/fpsyt.2022.734836)
Supplement: Supplementary file 1 [file Data_Sheet_1.docx]

**Search strategy**

**PubMed**

(((perinatal[Title/Abstract] OR mother[Title/Abstract] OR maternal[Title/Abstract] OR pregnancy[Title/Abstract] OR pregnant[Title/Abstract] OR postpartum[Title/Abstract] OR postnatal[Title/Abstract] OR prenatal[Title/Abstract] OR antenatal[Title/Abstract] OR antepartum)[Title/Abstract]) AND ((depression[Title/Abstract] OR depressive[Title/Abstract] OR mental health[Title/Abstract] OR mental disorders)[Title/Abstract])) AND ((Quality of Life[Title/Abstract] OR life quality[Title/Abstract] OR quality-of-life[Title/Abstract] OR HRQOL)[Title/Abstract])

Restricted on species (human), sex (female) and language (English and Chinese).

**Web of Science**

#1

AB=((perinatal OR mother OR  maternal  OR  pregnancy  OR  pregnant  OR  postpartum  OR

postnatal OR  prenatal  OR  antenatal  OR  antepartum)  AND  (depression OR depressive OR mental health OR mental disorders)  AND  (Quality of Life OR life quality OR quality-of-life OR HRQOL) )

#2

TI=((perinatal OR mother OR  maternal OR pregnancy OR pregnant OR postpartum OR postnatal OR prenatal OR antenatal OR antepartum) AND (depression OR depressive OR mental health OR mental disorders) AND (Quality of Life OR life quality OR quality-of-life OR HRQOL) )

#1 or #2

Restricted on language (English and Chinese).

**Embase**

(perinatal:ab,ti OR mother:ab,ti OR maternal:ab,ti OR pregnancy:ab,ti OR pregnant:ab,ti OR postpartum:ab,ti OR postnatal:ab,ti OR prenatal:ab,ti OR antenatal:ab,ti OR antepartum:ab,ti) AND (depression:ab,ti OR depressive:ab,ti OR 'mental health':ab,ti OR 'mental disorders':ab,ti) AND ('life quality':ab,ti OR 'quality of life':ab,ti OR hrqol:ab,ti) AND ([chinese]/lim OR [english]/lim) AND [female]/lim

**Scopus**

TITLE-ABS((perinatal OR mother OR maternal OR pregnancy OR pregnant OR postpartum OR postnatal OR prenatal OR antenatal OR antepartum) AND (depression OR depressive OR mental health OR mental disorders) AND (Quality of Life OR life quality OR quality-of-life OR HRQOL))

Restricted on language (English and Chinese)

**PsycINFO**

Abstract: perinatal OR Abstract: mother OR Abstract: maternal OR Abstract: pregnancy OR Abstract: pregnant OR Abstract: postpartum OR Abstract: postnatal OR Abstract: prenatal OR Abstract: antenatal OR Abstract: antepartum AND Abstract: depression OR Abstract: depressive OR Abstract: mental health OR Abstract: mental disorders AND Abstract: Quality of Life OR Abstract: life quality OR Abstract: quality-of-life OR Abstract: HRQOL

**Cochrane Central Register**

title abstract keyword (perinatal OR mother OR maternal OR pregnancy OR pregnant OR postpartum OR postnatal OR prenatal OR antenatal OR antepartum) AND (depression OR depressive OR mental next health OR mental next disorders) AND (Quality next of next Life OR life next quality OR quality-of-life OR HRQOL)

**The China National Knowledge Infrastructure**

(TKA=围产期 OR TKA=产妇 OR TKA=妊娠 OR TKA=孕妇 OR TKA=孕期 OR TKA=产前 OR TKA=产后 OR TKA=分娩前 OR TKA=分娩后 OR TKA=产褥期) AND (TKA=抑郁 OR TKA=精神 OR TKA=心理) AND (KY=生活质量 OR KY=生命质量OR KY=生存质量)

**The VIP Database for Chinese Technical Periodicals**

题目或关键词：围产期 OR 产妇 OR 妊娠 OR 孕妇 OR 孕期 OR 产前 OR 产后 OR 分娩前 OR 分娩后 OR 产褥期 与 题目或关键词：抑郁 OR 精神 OR 心理 与 题目或关键词：生活质量 OR 生命质量 OR 生存质量

**The Wan Fang Database for Chinese Periodicals**

题名或关键词:((围产期 OR 产妇OR 妊娠 OR 孕妇 OR 孕期 OR 产前 OR 产后 OR 分娩前 OR 分娩后 OR 产褥期) AND (抑郁 OR 精神OR 心理) AND (生活质量 OR 生命质量 OR 生存质量))
