# Supplementary material for: Quality of Life in Mothers With Perinatal Depression: A Systematic Review and Meta-Analysis
Source: Front Psychiatry. 2022 Feb 15;13:734836. doi: 10.3389/fpsyt.2022.734836 (PMC8886107; doi:10.3389/fpsyt.2022.734836)
Supplement: Supplementary file 2 [file Data_Sheet_2.docx]

**Quality assessment**

**Cohort study**

| Study | Representativeness of the exposed cohort | Selection of the non exposed  cohort | Ascertainment of exposure | Outcome was not present at start of study | Comparability | Assessment of outcome | Follow-up time | Adequacy of follow up of cohorts | Total  scores | Kappa |
| --- | --- | --- | --- | --- | --- | --- | --- | --- | --- | --- |
| Sadat2014 | 1* | 1 | 1 | 1 | 2 | 1 | 1 | 1 | 9 | 89% |

Note:* score of each question

**Case control studies**

| Study | Adequate definition of cases | Representativeness of the cases | Selection  of controls | Definition  of controls | Comparability | Ascertainment  of exposure | Same method for cases and controls | Non-Response rate | Total  scores | Kappa |
| --- | --- | --- | --- | --- | --- | --- | --- | --- | --- | --- |
| Abbaszadeh2013 | 1* | 1 | 1 | 1 | 2 | 1 | 1 | 1 | 9 | 89% |
| Qiu2015 | 1 | 0 | 1 | 1 | 2 | 1 | 1 | 1 | 8 | 89% |

Note:* score of each question

**Cross sectional studies**

| Study | Inclusion criteria  defined clearly | Study subjects  and the setting described in detail | Exposure measurement | Condition  measurement | Confounding factors identification | Treatment of confounding factors | Outcome  measurement | Statistical analysis | Kappa |
| --- | --- | --- | --- | --- | --- | --- | --- | --- | --- |
| Costa2006 | Yes | Yes | Yes | Yes | Yes | Yes | Yes | Yes | 100% |
| Tychey2007 | No | Yes | Yes | Yes | Yes | Yes | Yes | Yes | 100% |
| Nicholson2006 | Yes | Yes | Yes | Yes | Yes | Yes | Yes | Yes | 100% |
| Tungchama2017 | Yes | Yes | Yes | Yes | Unclear | Unclear | Yes | Yes | 100% |
| Chen2013 | Yes | No | Yes | Yes | Unclear | Unclear | Yes | Yes | 100% |
| Hu2017 | No | Yes | Yes | Yes | Unclear | Unclear | Yes | Yes | 100% |
| Li2011 | Yes | Yes | Yes | Yes | Unclear | Unclear | Yes | Yes | 100% |
| Zhang2008 | No | Yes | Yes | Yes | Unclear | Unclear | Yes | Yes | 100% |
| Tsai2018 | Yes | Yes | Yes | Yes | Yes | Yes | Yes | Yes | 100% |
