# Supplementary material for: Quality of Life in Mothers With Perinatal Depression: A Systematic Review and Meta-Analysis
Source: Front Psychiatry. 2022 Feb 15;13:734836. doi: 10.3389/fpsyt.2022.734836 (PMC8886107; doi:10.3389/fpsyt.2022.734836)
Supplement: Supplementary file 3 [file Data_Sheet_3.docx]

**Funnel plots**

1. Bodily pain

1. General health

1. MCS (Mental component score)

1. Mental health

1. PCS (Physical component score)

1. Physical functioning

1. Role-Emotional

1. Role-Physical

1. Social functioning

1. Vitality
